# Overview of Paraneoplastic Autoantibody-Mediated Cognitive Impairment and Behavioral Changes: A Narrative Review

**DOI:** 10.7759/cureus.51787

**Published:** 2024-01-07

**Authors:** Duaa Alkhayat, Zakaria Y Khawaji, Amal M Sunyur, Omnyah A Sanyour, Ahmad S Badawi

**Affiliations:** 1 Clinical Oncology, Taibah University, Medina, SAU; 2 Medicine and Surgery, Taibah University, Medina, SAU; 3 Psychiatry, King Salman Medical City, Medina, SAU

**Keywords:** autoantibody behavioral change, autoantibody cognitive disturbance, paraneoplastic psychiatric syndromes, paraneoplastic encephalitis, paraneoplastic encephalitis syndromes, paraneoplastic behavioral change, paraneoplastic cognitive dysfunction, paraneoplastic autoantibodies, paraneoplastic neurological syndrome, paraneoplastic

## Abstract

Cognitive dysfunction and behavioral change can be some of the manifestations of cancer, occurring as a part of paraneoplastic neurological syndrome, most commonly in small cell lung cancer. Paraneoplastic limbic encephalitis is the leading cause of cognitive disturbance and abnormal behavior in paraneoplastic syndromes, which is usually autoantibody-mediated. Autoantibodies are the main contributors to the development of cognitive dysfunction and behavioral change in cancer patients, with studies suggesting a higher liability for antibody-positive cancer patients to be affected. Anti-NMDAR and anti-AMPAR are antibodies targeted against surface antigens, manifesting predominantly as memory disturbance, abnormal behavior, psychiatric symptoms, and seizures. Other surface antigen-targeted antibodies include anti-GABA, anti-CASPR2, and anti-LGI1, which were shown to have cognitive function impairment and abnormal behavior as some of the main presentations, predominantly affecting memory. Cognitive deterioration and changes in behavior were also relatively common with some of the intracellular antigen-targeted antibodies, including anti-Hu, anti-SOX1, anti-PCA2, and anti-Zic2. Affected behavior and cognition, however, were reported less commonly in other paraneoplastic antibodies against intracellular antigens (anti-Yo, anti-GAD, anti-Ma2, anti-Ri, anti-CV2, and anti-KLHL11). Our article will provide a comprehensive review of the clinical manifestations of cognitive impairment and behavioral changes among cancer patients who develop paraneoplastic syndrome. Additionally, this review will discuss the role of specific paraneoplastic autoantibodies and the clinical spectrum linked to each separately.

## Introduction and background

Paraneoplastic syndrome encompasses a group of heterogeneous clinical syndromes that are attributed to the remote indirect effects of tumors. It is believed to be caused by the secretion of functional peptides and hormones by the tumor or the immune cross-reactivity response [1]. These distinct effects of malignancy can impact various systems, most commonly the endocrine and nervous systems [2]. Paraneoplastic syndromes are one of the causes of higher cortical function disturbances, which usually include cognition, behavior, and affection [3]. Generally, nervous system affection occurring secondary to a distant tumor is known collectively as paraneoplastic neurological syndromes (PNS). This term, however, includes cognitive and behavioral symptoms along with other neurological manifestations, such as seizures, neuropathies, or myopathies. Central PNS can manifest differently; common examples are limbic or brainstem encephalitis, encephalomyelitis, or cerebellar degeneration. Although studies on the epidemiological features of PNS show that its incidence is somewhere between one and eight per 100,000, it is thought that the rates can be as high as one per 300 patients with tumors, which is suggestive of a possibly understudied area [4].

Cancer patients, including those in remission, may experience cognitive challenges impacting daily life, work, and overall well-being. Factors contributing to cognitive impairment in patients with cancer can stem from cancer itself (like those related to central nervous system (CNS) tumors or paraneoplastic syndrome), concurrent conditions (depression, anxiety, fatigue), or treatment-related cognitive effects like the ones observed in patients treated by chemotherapy, radiotherapy, or hormonal therapy [5]. When assessing cancer-related cognitive issues, it is crucial to prioritize tests gauging frontal-subcortical network functions like learning, memory, executive functioning, processing speed, and motor coordination, as these cognitive domains were found to be more significantly impaired in patients with cancer, especially those receiving chemotherapy [6].

In October 2006, the International Cognition and Cancer Task Force (ICCTF) was established to enhance the comprehension of how cancer and its treatments affect cognitive and behavioral functions in adults with non-CNS cancers [7]. The ICCTF, which utilizes objective neuropsychological tests, is considered the gold standard for assessing cognitive function when evaluating the cognitive impact of cancer and its treatments. The ICCTF formed two working groups, which issued recommendations for standardized criteria to define cognitive impairment and changes, along with specific proposals for a core set of cognitive tests to assess the cognitive function of cancer patients (Table 1) [8].

**Table 1:** The International Cognition and Cancer Task Force criteria to define cognitive impairment and the proposed test to evaluate cancer patients’ cognitive function. This table was adapted from [8].

Core assessment		
Recommended cognitive domain	Recommended cognitive assessment measures	Specific cognitive skills evaluated
Learning and memory	Hopkins Verbal Learning Test-Revised (HVLT-R)	Verbal learning and memory assessed by list learning, immediate recall, delayed recall, and recognition
Executive function	Trail Making Test	Multiple cognitive skills involved in performing tasks, including attention, working memory, information processing speed, and mentality flexibility
	Controlled Oral Word Association (COWA) (also known as FAS test)	Spontaneous generation of words
Processing speed	Trail Making Test	Multiple cognitive skills involved in performing tasks, including attention, working memory, information processing speed, and mentality flexibility
Supplemental assessment		
Recommended cognitive domain	Recommended cognitive assessment measures	Specific cognitive skills evaluated
Working memory	Auditory Consonant Trigrams (ACT)	Short-term or working memory task requiring online maintenance of information while performing an interference task during a delay
	Paced Auditory Serial Attention Test (PASAT)	Serial attention task assessing working memory, divided attention, and information processing speed
	Brief Test of Attention (BTA)	Auditory divided attention
	Wechsler Adult Intelligence Scale-IV (WAIS-IV) Letter Number Sequencing	Working memory, attention, and mental control

The ICCTF emphasizes a collaborative approach that integrates data across studies, employs shared definitions, and suggests a core set of cognitive tests for distinct cognitive domains to offer "best practice" guidance [8].

Our aim in this narrative review is to provide an overview of the paraneoplastic neuronal autoantibodies that are implicated in the development of cognitive dysfunction and behavioral change in cancer patients. This comes as an attempt to facilitate further research on this subject to expand our understanding of it. Articles were searched for mainly in Google Scholar and PubMed. Articles that were more updated and which were conducted in more reliable study designs (according to the hierarchy of evidence) were chosen over the ones that were older or had less reliable designs. The most updated articles on a certain topic and those with the best study design were therefore included.

## Review

PNS have been associated with anti-neuronal autoantibodies, which were further classified according to the targeted antigen into intracellular-directed antibodies, which are directed to antigens that are found on the inside of a neuron, or surface antibodies, which are directed toward antigens that lie on the surface of the neurons [4]. The most frequent antibody in PNS is the anti-Hu antibody, which is known to cause several syndromes, including limbic encephalitis, cerebellar degeneration, and neuropathies [9]. Limbic encephalitis is the most common presentation of paraneoplastic syndrome leading to cognitive and behavioral impairment, often called paraneoplastic limbic encephalitis (PLE). PLE can occur in patients with a prior diagnosis of cancer (known cancer) or those with occult tumors [10].

Lung cancer is one of the most commonly associated tumors with PNS, and it was found to have a high prevalence of neuronal antibodies in a retrospective study done in 2017 [11]. In a cross-sectional study done in 2021, 45% of small cell lung cancer (SCLC) and 34% of non-small cell lung cancer (NSCLC) patients were positive for neuronal antibodies, which had increased rates of cognitive dysfunction when compared to those who were negative for autoantibodies [12]. In another study, an investigation of cognitive dysfunction among patients with melanoma was done. Melanoma patients who were antibody-positive were three times more likely to develop cognitive impairment than those who were antibody-negative. Furthermore, antibody-positive patients demonstrated significantly impaired overall cognitive function [13]. Examples of other tumors that could result in PNS are breast cancer, prostate cancer, ovarian teratoma, neuroendocrine tumors, testicular seminoma, Hodgkin’s and non-Hodgkin’s lymphomas, and thymomas [8].

It has been suggested that the affection of the CNS results from the intrathecal synthesis of antibodies, which may be caused by a translocation of lymphocytes into the cerebrospinal fluid (CSF). Major histocompatibility complex (MHC) molecules have also been suggested to be another factor contributing to the development of PNS, either by increased expression of the molecule or mediated through human leukocyte antigen (HLA)-DQ2 and HLA-DR3 class II molecules [9]. Antibodies directed against intracellular proteins are thought to cause the disease through T-cell-mediated cytotoxicity. Whereas antibodies against surface proteins or receptors are thought to have antibody-mediated pathogenesis [14].

Paraneoplastic antibodies targeting surface antigens

*Anti-N-Methyl-D-Aspartate Receptor* *(NMDAR) Antibody*

Glutamate, along with gamma-aminobutyric acid (GABA), are two neurotransmitters that are crucial for cognitive function. The N-methyl-D-aspartate (NMDA) receptor (NMDAR) is an important contributor to the excitatory function of glutamate, which in turn impacts learning and memory processes. Therefore, changes in the function of these receptors impact neuronal signaling and cognitive processes. NMDAR antibodies result in the internalization of the receptors into the cell by binding to the NR1 subunit. This, in turn, causes a decrease in the density of the receptors on the neuronal surface and, therefore, a reduction in NMDA-dependent signaling. The frontal cortex and hypothalamus contain NMDARs in high concentrations. This might explain the underlying cause of the impairment of memory and executive function associated with NMDAR antibody syndromes. As much as the process of internalization of NMDARs caused by NMDA antibodies is reversible, several consequences can be irreversible, like damage to the superficial white matter (which contributes to the dysfunction of attention and memory) and the structural damage of the hippocampus [15].

Anti-NMDAR antibody-associated encephalopathy occurs commonly as an autoimmune disorder. However, paraneoplastic production of the antibodies is not uncommon. Ovarian teratomas are the most commonly associated tumor with anti-NMDAR antibodies, usually in a young female. However, it can also occur with SCLC and breast cancer, but usually in older age groups. In young males, the syndrome is usually attributed to testicular teratomas, which may be coexistent with anti-Ma1/Ma2 antibodies [16].

In the acute phase, the clinical manifestations of anti-NMDAR encephalopathy are predominated by psychosis, cognitive dysfunction, and several neurological symptoms, including seizures, movement disorders, autonomic instability, and loss of consciousness. Furthermore, patients may exhibit symptoms of behavioral and personality change, mood liability, paranoia, fear or anxiety, blunted affect, asocial behavior, and unusual aggression. Catatonia, central hypoventilation, and depression are also among the symptoms that could be caused by anti-NMDAR encephalitis. The onset of symptoms might be preceded by a period of flu-like prodrome, manifesting as headaches and fever [10,15-17].

Cognitive disturbance in the acute phase is often severe and involves all domains of cognition. Dysfunctions in memory and executive function are the most pronounced disturbances in cognition, but other domains are also affected in varying degrees [18,19]. Case reports, however, have reported unusual presentations of cognitive disturbances, including age awareness disturbance, transient epileptic amnesia, and a fluctuation of cognitive capacity that is concurrent with visual hallucinations and sleep disturbance, which was misdiagnosed as Lewy body dementia [20-22].

As regards the long-term cognitive affection of anti-NMDA receptor encephalitis, the same extensive cognitive affection occurring in the acute phase can persist with the patient for years. However, episodic memory and executive function, just like in the acute phase, remain the most affected cognitive functions after one year of the initial insult [18,19,23-25].

Anti-Alpha-Amino-3-Hydroxy-5-Methyl-4-Isoxazole Propionic Acid Receptor (AMPAR) Antibody

The AMPA receptor is a tetrameric ligand-gate that is composed of a combination of Glu subunits (GluA1-4). The AMPA receptor drives the majority of fast-excitatory glutaminergic transmission. Upon binding glutamate, the AMPA receptor is activated, allowing the influx of cations (predominantly sodium and calcium ions), which results in post-synaptic neuron depolarization. It is important to note that AMPA receptors are essential for two complementary processes known as "long-term potentiating (LTP)” as well as "long-term depression (LTD)”. LTP is a neuroplasticity process characterized by a persistent increase in the strengthening of synapse transmission following stimulation of post-synaptic neurons. On the other hand, LTD is the reversal with a notable reduction of synaptic connection efficacy after a long stimulus [26-28]. Synaptic plasticity is widely implicated in the hippocampus, and thus its importance could be demonstrated in cognitive, learning, and memory functions [29].

AMPAR encephalitis is a rare entity of autoimmune encephalitis, and more than half of patients are diagnosed with limbic encephalitis (LE). Typically, it manifests as an acute or subacute onset with impairment of short-term memory, disorientation, aberrant behavior, and seizures. The autoantibodies are targeted largely against GluA1-2 subunits, thereby disturbing the extracellular portion of the AMPA receptors. Decreased AMPAR density will ultimately result in a remarkable inhibition of synaptic transmission in favor of intrinsic excitation. This alteration of AMPAR trafficking initiated by antibodies is the uttermost player in the induction of short-term memory loss and seizures, among other clinical features. A recent review in 2021 conducted a thorough search of AMPA encephalitis cases that were published in the literature. About 60.6% of all reported cases were associated with detected tumors or a history of tumors suggesting a paraneoplastic origin. It was found that thymoma is the most frequent concomitant cancer among patients with AMPAR encephalitis, and most of them are younger than 60 years of age. SCLC, breast cancer, and ovarian cancer were also reported [30].

The first clinical analysis of AMPAR LE was conducted in 2009. Ten patients were enrolled in the study, and seven had lung, breast, and thymus tumors. AMPAR antibodies were detected in all patients. Most of the patients presented with classic LE manifestations with a subacute onset of memory loss, confusion, and disorientation. Progressive dementia with behavioral changes, agitation, and marked memory loss was reported in one patient. Seizure was also seen in four patients [31]. Apart from LE typical cognitive features, patients with AMPAR antibodies may present with atypical clinical manifestations that indicate limbic dysfunction with diffuse encephalopathy or a prominent psychosis with bipolar features. Also, prominent psychiatric manifestations, abnormal behavior, and visual hallucinations were seen [32]. Despite the usual subacute onset, patients may have an acute onset of cognitive decline with anterograde amnesia, executive dysfunction, and confusion. Fulminant encephalitis is an unusual AMPAR antibody-associated disorder [33]. As expected, the most prominent cognitive dysfunction in all patients with AMPAR LE is a motor deficit, especially short-term memory [31-36]. Psychodynamic syndromes with mood disorders, anxiety, and suicidal thoughts were seen in one female patient with ovarian cancer [34]. The typical symptoms of positive AMPAR autoimmune encephalitis with abnormal behavior, mainly agitation and forgetfulness, have been reported in a case report of medullary thyroid cancer [35,37].

In several case reports, a set of behavioral changes were observed. Progressive behavioral changes include eating soap, irritability and agitation, slowed thinking (bradyphrenia), failure to recognize family members, repetitive stereotyped words and behaviors, and decreased social interaction. All patients have memory impairment as a cardinal feature of paraneoplastic AMPAR encephalitis [38-40].

The long-term outcomes of AMPA encephalitis, regardless of the brain site, largely rely on the proper treatment of the oncological condition. Partial or even complete recovery and impressive progress in improving cognitive function are possible with the early initiation of immunotherapy and conventional therapy for underlying cancer [32,34-37].

Anti-Gamma-Aminobutyric Acid (GABA) Receptor Antibodies

In contrast to glutamate, gamma-aminobutyric acid A (GABA) is the major inhibitory neurotransmitter in the CNS. Ionotropic GABAA and metabotropic GABAB are crucial receptors for mediating GABA neurotransmitter efficacy. GABA action by inhibiting neuron signaling propagation depends on the post-synaptic neurons through fast hyperpolarization of GABAA receptors by chloride (Cl-) ion influx. On the other hand, GABAB receptors are responsible for the slow inhibition of transduction. Activation of these receptors will initiate an intracellular signaling cascade, which in turn results in activation of post-synaptic potassium receptors or inhibition of presynaptic calcium receptors. GABAergic transmission is critical for the orientation of sensory inputs. The correlation between GABA activity and cognitive functions is yet to be well understood. However, recent studies suggest the important role of GABA in several cognitive domains, including working memory, impulsivity, motor distraction, motor learning processing, and executive functions [41-43].

GABAB receptor-antibody encephalitis is a recently identified type of autoimmune encephalitis that involves the limbic system. In this condition, the antibodies are targeted against the B1 subunit, which hinders the function of GABAB channels. The main clinical manifestations include seizures, cognitive dysfunctions, and behavioral changes. The paraneoplastic GABAB antibodies are mostly associated with lung cancer [15,44]. A case series of 20 patients, half of whom were diagnosed with SCLC, found that the majority of subjects exhibited memory loss, personality changes, confusion, and hallucinations. These features are consistent with classical LE [45]. Several case series and reports have described the presentation of patients with positive GABAB antibodies. While cognitive and behavioral dysfunction is generally reported, there is a lack of information about the specific cognitive domains affected in the majority of the studies. Nevertheless, short-term memory deficits appear to be the most prominent symptom. Confusion, disorientation, poor spatial-temporal orientation, dysexecutive syndrome, unresponsiveness, and a sluggish thought process are among the features of cognitive decline associated with GABAB antibodies. To a lesser extent, some cases may present with abnormal behaviors and psychiatric manifestations, including aggressiveness and irritability, nonsensical talking (balderdash), restlessness, hallucinations, personality changes, mood liability, and sleep disorders [43,46-51].

Paraneoplastic GABAB-antibody encephalitis has worse outcomes than AMPAR LE, with severe brain damage, serious complications, and high mortality rates. Certain factors have been identified as significant predictors of long-term outcomes. Older age, alteration in consciousness, concomitant malignancy, multiple anti-neuronal antibodies, more pronounced clinical manifestations, and hyponatremia are correlated with poor outcomes [50,51].

Compared to GABAB receptor antibodies, GABAA receptor antibodies are less frequent and usually affect a younger population. Of all reported cases, only 20% of patients had tumors, with thymoma being the most common association [52]. GABAA receptor encephalitis is known to cause eminent epileptic attacks with multifocal brain MRI abnormalities. The behavioral abnormalities and cognitive impairment features include short-term memory loss, confusion, psychosis, personality changes, apathy, irritability, and insomnia. Generally, most patients respond well to immunotherapy with favorable outcomes [53-55].

Anti-Voltage-Gated Potassium Channel (VGKC)-Associated Proteins Antibodies

Voltage-gated potassium channel (VGKC)-associated proteins are contactin-associated protein-like 2 (CASPR2) and leucine-rich glioma-inactivated protein (LGI1). CASPR2 is a surface protein expressed in the CNS and peripheral nerves. This surface protein is important for the proper functioning and localization of VGKC [56]. CASPR2 antibodies are mostly associated with thymomas, most likely in old men. In patients with paraneoplastic anti-CASPR2 antibodies, Morvan’s syndrome is likely to be a predominant clinical feature, but other manifestations like limbic encephalitis, neuromyotonia, cerebellar syndrome, or painful peripheral nerve syndrome can also occur. Interestingly, paraneoplastic patients with concurrent thymoma usually have worse outcomes than nonparaneoplastic patients [4]. Manifestations of patients with CASPR2 antibodies include cognitive deficits as the main symptom for these patients, which mainly present as memory disturbance. Also, behavioral disturbance and psychotic symptoms (hallucinations, delusions) are major features. Other neurological manifestations may include seizures, cerebellar dysfunction, neuropathies, hyperhidrosis, and dysautonomia [56].

As for LGI1, it is reported in cases of malignancy of the lung, thyroid, breast, kidney, prostate, ovarian teratomas, and thymomas. It is known to be a synaptic protein that is related to VGKC [57]. In a systematic review that involved 485 subjects, 412 people with anti-LGI1 had cognitive impairment. The majority of these patients had short-term memory loss; less common cognitive symptoms included disorientation, language dysfunction, executive function disturbance, or visuospatial dysfunction; and only a few had inattention, disturbed calculation, or impaired comprehension. Furthermore, psychiatric symptoms like emotional instability, aberrant motor behavior, apathy, agitation, hallucinations, delusions, disinhibition, anxiety, depression, or personality change were also reported. Other symptoms included mainly neurological symptoms like seizures, dysautonomia, and movement disorder [58].

A recent systemic review and meta-analysis of 32 studies examined the neurological and psychiatric manifestations of patients with LGI-1 and CASPR limbic encephalitis. In all seven studies involving patients with anti-LGI1, cognitive impairment was observed in all patients, as shown by the Montreal Cognitive Assessment (MoCA) scores. Four studies reported severe cognitive deficits, two showed MoCA scores suggestive of moderate cognitive decline, and one study reported mild cognitive impairment. Additionally, nonverbal short-term and working memory scores were abnormal, with delayed free memory recall. However, verbal memory (both working and short-term memory) scores fell within the normal range in the studies included. In regard to executive functions and attention, studies utilizing a formal scoring system yielded normal results, indicating the absence of dysexecutive syndrome. These executive functions that were assessed include reasoning, word fluency, social-emotional skills, and processing speed. Only two patients with anti-CASPR autoimmune LE were included in the systemic review, and their cognitive functions, apart from memory function, were not formally assessed. Both patients showed a memory deficit before the initiation of immunotherapy [59].

Paraneoplastic antibodies targeting intracellular antigens

Anti-Hu Antibody

The anti-Hu antibody, which is also known as anti-neuronal nuclear antibody 1 (ANNA-1), is an antibody targeted against a group of intracellular proteins known as Hu proteins. This antibody recognizes a group of genes, HuA, HuB, HuC, and HuD, which are thought to affect mRNA function within neurons. It is still not known whether these antibodies result in the development of the disease or result from it, with newer evidence suggesting that the disease results from disordered T-cell activity, which might be activated by the Hu proteins. Despite that, the exact mechanism by which it disrupts the function of this protein remains poorly understood up to this moment [60-62].

Anti-Hu antibodies usually occur in males who are around the age of 60. Anti-Hu antibodies have a high oncological association, with tumor frequency in almost all cases. Paraneoplastic anti-Hu antibodies are mainly associated with SCLC, but they can be present with other types of lung cancer or with breast, gastrointestinal, ovarian, prostate, or neuroendocrine malignancies. Anti-Hu predominantly causes sensory neuropathy; however, it may also result in cerebellar degeneration and may cause limbic or cortical encephalitis, which is the main cause of cognitive and behavioral symptoms in these patients. Rhombencephalitis, dysautonomia, and sensorimotor neuropathies are also caused by anti-Hu antibodies [4].

Anti-Hu antibodies target the hippocampus and thalamus, which are the regions responsible for regulating fear responses, emotions, memory, and sensorimotor functions. Thus, patients may manifest with sudden personality changes and memory impairment [63]. In a case series of 71 patients, cognitive symptoms of anti-Hu included confusion and disorientation, affect disruption, cognitive decline, and short-term memory loss. Hallucinations are also among the reported psychiatric symptoms of anti-Hu [64].

Anti-Yo Antibody

Anti-Yo, or Purkinje cell cytoplasmic antibody type 1 (PCA1), is an antibody directed against a cytoplasmic neural protein known as cerebellar degeneration-related protein 2 (CDR2). The mechanism by which it causes disease is still vague; however, it has been suggested that cytotoxic T-cells play a vital role in the pathogenic mechanism, resulting in the destruction of Purkinje cells of the cerebellum [65].

Paraneoplastic anti-Yo antibody production usually occurs in females with a median age of 60. It has a high paraneoplastic association in about 90-100% of cases. Paraneoplastic anti-Yo antibody production usually occurs in gynecological malignancies (ovarian or tube malignancies) and breast cancers. However, it can rarely occur in men (upper GI adenocarcinoma or prostate cancer). Anti-Yo mainly causes paraneoplastic cerebellar degeneration (PCD) but can also cause peripheral neuropathies [4]. One retrospective study published in 2006 found that the rates of anti-Yo antibody positivity in 557 ovarian cancer patients were 2.3% and 1.6% in 253 breast cancer patients. However, only 12% of patients with anti-Yo antibodies had PCD [66].

Patients with anti-Yo usually manifest severe cerebellar symptoms, including ataxia involving both the trunk and limbs, diplopia, dysarthria, and nystagmus, as a result of cerebellar degeneration [67]. Therefore, cognition is usually unaffected. However, some reports have reported a 20% rate of mild cognitive impairment and memory deficits in patients with PCD [68]. Furthermore, several studies reported cases of anti-Yo associated with cognitive-affective syndrome, which results in defective executive function, visuospatial function, and language, along with behavioral disinhibition. This usually occurs secondary to limbic encephalitis, which occurs concurrently with cerebellar degeneration in anti-Yo antibodies [69-71].

Anti-Sry-Like High Mobility Group Box (SOX1) Antibody

Because of their association with neoplastic disorders, anti-Sry-like high mobility group box (SOX1) antibodies are partially classified as neural autoantibodies. Anti-SOX1 antibodies have been linked to a number of clinical presentations, such as PCD and Lambert-Eaton myasthenic syndrome (LEMS). The proteins that are related to SOX1 have been identified as the antigens that trigger the immune response. Developmental transcription factors known as SOX1 proteins play a role in the proper development of the CNS and share a common DNA-binding protein. Anti-SOX1 antibodies have been linked to several neurological disorders, with LEMS being the most common clinical feature. In addition to LEMS, reports of PCD, PLE, and neuropathy have also been made. Because of their strong association with tumors, anti-SOX1 autoantibodies are thought to be onconeural autoantibodies associated with malignant neoplasms, although their exact etiology is still unknown.

The most associated tumor with PNS is SCLC, which is estimated to be present in 3-5% of PNS cases. Similarly, anti-SOX1 antibodies are found in up to 36.5% of SCLC patients and are similarly thought to be serological indicators of the disease. Furthermore, it has been shown that in 49% of patients with PCD and SCLC, anti-SOX1 antibodies are the most often found autoantibodies. Research performed over the last 15 years has demonstrated that individuals with anti-SOX1 antibodies have PCD. Additionally, we have detected chronic PCD in patients with NSCLC that has mediastinal squamous-cell carcinoma and anti-SOX1 antibodies.

PCD in anti-SOX1 antibodies is thought to be caused by the SOX1 antigen and related proteins that have accumulated in the Purkinje cell layer of the adult human cerebellum, while the exact mechanisms underlying this condition are still unknown. Anti-SOX1 antibody patients present with a broader spectrum of neurological symptoms than is usually recognized, and they may be mistakenly diagnosed as neurodegenerative diseases. Multiple levels of neurexins, including the limbic system, cerebellum, peripheral nervous system, and neuromuscular junction, may be involved in the neurological dysfunction linked to anti-SOX1 antibodies.

Memory and cognitive deficits with acute or subacute onset are among the clinical hallmarks of LE. Confusion, psychiatric symptoms (such as anxiety, depression, or psychosis), behavioral abnormalities, seizures, and movement disorders (such as ataxia) are some additional symptoms [72].

Anti-Ma2 Antibody

The typical clinical phenotype linked to Ma2 IgG is paraneoplastic limbic and/or diencephalic encephalitis. In addition, some of these patients exhibit secondary narcolepsy. Anti-Ma2 antibodies have a strong correlation with NSCLC in elderly patients who also have co-existing Ma1 IgG and testicular tumors in young men, which are typically non-seminomatous germ cell tumors also present in elderly patients with NSCLC and breast cancer. If a man presents with LE and is younger than 50 years, it should be suspected. The diencephalon, upper brainstem, and limbic system are among the CNS regions that are impacted. Individuals may present with hypokinesis, limb rigidity, vertical gaze palsy, and orofacial and jaw dystonia. Sleep abnormalities can manifest as cataplexy, narcolepsy, excessive daytime sleepiness, and rapid eye movement sleep disorders [10,14].

Rarely occurring anti-Ma2 antibody encephalitis is characterized by ataxia, seizures, dysarthria, reduced mentality, and neuro-ophthalmologic abnormalities. Additionally, brainstem, hypothalamus, and cognitive symptoms occur as a result of LE. Compared to other reports, in one case report, a patient presented with dominant psychiatric symptoms, including aggression, disorganized language, depression, anxiety, and memory impairment; however, there was no evidence of seizures, ataxia, neuro-ophthalmologic abnormalities, or systemic tumors. Therefore, although the patient manifested unusual symptoms, he did not visit the hospital for two months because his family assumed that he was experiencing psychiatric problems (e.g., depression, stress, and anxiety). His inflammatory brain lesion might have slowly progressed; his early psychiatric symptoms were consistent with the initial MRI finding of damage to the right medial temporal lobe and hippocampus [73].

Anti-CV2 Antibody

Also known as the collapsin response-mediator protein-5 (CRMP5) antibody, it is an IgG that has been described in association with paraneoplastic peripheral neuropathy, cranial neuropathy, gastroparesis, encephalitis, cerebellar ataxia, myelopathy, and chorea [14].

Individuals with paraneoplastic syndrome produce several antibodies, each of which targets a distinct antigen and results in distinct symptoms and signs. One of the main antibodies of this type is the CV2/collapsin response mediator protein antibody. It causes damage to the nervous system, which frequently shows up as peripheral neuropathy, myelopathy, ocular manifestation, chorea, limbic encephalitis, and cerebellar ataxia. The clinical diagnosis of PNS depends on the detection of the CV2/CRMP5 antibody. Immunological and anti-tumor therapies can help improve prognosis and reduce symptoms [74].

When frontostriatal and basal ganglia circuitry are involved, encephalomyelitis that rarely affects only the limbic system causes cognitive and behavioral deficits in patients with cognitive symptoms. Cerebellar degeneration, chorea, uveitis, optic neuritis, myelitis, and peripheral neuropathy are other presentations [10].

Anti-Neuronal Nuclear Antibody Type 2 (ANNA-2, Anti-Ri)

Anti-Ri, also called anti-neuronal nuclear antibody-2 (anti-ANNA-2), antibodies most commonly result in opsoclonus-myoclonus (involuntary, arrhythmic, chaotic, multidirectional saccades with horizontal, vertical, and torsional components) [10] as well as cerebellar ataxia in breast cancer patients. A recent French study also supported the high breast cancer association. There was greater variation in the types of cancer found in men, with bladder and lung cancer being the most frequently detected. The most typical neurological presentations are brainstem and cerebellar syndromes. There have also been reports of myelopathies, cranial and peripheral neuropathies, and encephalitis, with or without seizures. It has been reported that up to 25% of patients with ANNA-2 paraneoplastic encephalitis have laryngospasm and/or jaw dystonia [14].

Anti-Purkinje Cell Antibody Type 2 (PCA-2) Antibody

Also known as microtubule-associated protein 1B (MAP1B), it is associated with peripheral neuropathy, which is emerging as one of the most common clinical phenotypes [75]. Notably, a substantial proportion of peripheral neuropathy cases exhibit concurrent CNS involvement, often linked to SCLC. Remarkably, PCA-2 is highly specific to the presence of SCLC [76].

This antibody has been associated with a spectrum of manifestations, including polyradiculoneuropathy, cerebellar ataxia, encephalitis, motor neuropathy, dysautonomia, and LEMS. Cognitive symptoms, or encephalopathy, result from cortical affection. Limbic encephalitis is also possible, causing behavioral changes and a memory deficit. Hallucinations, personality changes, and paranoia were also reported in some patients. Sleep disturbance was also reported in patients with diencephalic dysfunction [75].

Anti-Kelch-Like Protein 11 (KLHL11) Antibody

KLHL11 is a recently discovered onconeural antibody associated with the development of rhombencephalitis, which manifests in the brainstem and/or cerebellum. Additionally, KLHL11 has been linked to reported cases of limbic encephalitis, either with or without concurrent rhombencephalitis. Testicular germ cell tumors are the most common oncological association. Frequent neurological presentations encompass rhombencephalitis, along with ataxia, diplopia, vertigo, and auditory symptoms (hearing loss and tinnitus), with tinnitus often presenting early. Other manifestations include dysarthria and seizures. Neuropsychiatric dysfunction can manifest in these patients too, causing anxiety and panic attacks or cognitive decline [77].

Anti-Glutamic Acid Decarboxylase (GAD) Antibody

GAD65 is a pyridoxal 5'-phosphate-dependent enzyme, expressed widely in the CNS and pancreatic β-cells. Its role involves catalyzing the conversion of the excitatory neurotransmitter l-glutamate to the inhibitory neurotransmitter GABA [78]. Recent studies indicate that monoclonal GAD65 antibodies can disrupt GABAergic neurotransmission, leading to neurophysiological and behavioral effects resembling cerebellar ataxias [79].

Anti-Zic2 Antibody

Zinc finger proteins, encoded by Zic genes and expressed in both the mature and developing CNS, contribute significantly to cerebellar development [80]. The association of Zic proteins with cerebellar development implies that autoantibodies targeting these proteins can lead to significant cerebellar effects. Autoantibodies targeting these proteins, particularly Zic4, are associated with significant cerebellar effects, as seen in cases of cerebellar dysfunction reported by Sabater et al. [81].

Salazar et al. identified Zic4 antibodies alongside 14-3-3 antibodies in a reported case exhibiting rapidly progressive dementia, stroke-like symptoms, and dysautonomia, eventually diagnosed as possible Creutzfeldt-Jakob disease. Notably, he suggested that the rapid destruction in the CNS could potentially be attributed to anti-Zic4 antibodies [82].

SCLC is the predominant tumor type associated with Zic4 antibodies, found in about 90% of cases. PCD is the most common syndrome in both isolated Zic4 cases and those with additional onconeural antibodies [83].

In cases of rapidly progressive dementia, a diagnosis of autoimmune encephalitis should be considered, presenting with cognitive decline, seizure-like activity, and newly emerging movement disorders [84]. Although Zic4 antibodies are mainly linked to SCLC, there are reports of associations with other malignancies like B-cell lymphoma and neuroblastoma [80].

Study implications and recommendations

This article provides an overview of the available evidence on the relationship between cognitive dysfunction and paraneoplastic syndrome, which will help establish the foundation for further clinical research, with a focus on early identification and proper intervention if necessary. More extensive research should be conducted on this topic to enhance our understanding of the factors contributing to cognitive dysfunction and behavioral change in cancer patients so that early interventional measures can be implemented into the treatment plan.

## Conclusions

In this study, we found that patients who were positive for paraneoplastic autoantibodies were more likely to develop cognitive impairment and behavioral change than those who were autoantibody negative. Furthermore, we found that anti-NMDAR, anti-AMPAR, anti-GABA, anti-CASPR2, anti-LGI11, anti-Hu, anti-Zic2, anti-SOX1, and anti-PCA2 antibodies were more commonly associated with cognitive deterioration (primarily memory deficits), behavioral change, and psychiatric symptoms. However, these were reported less commonly in other antibodies.
